# Morphological, vascular, and functional changes in macular telangiectasia type 2 in Egyptian patients

**DOI:** 10.1038/s41598-025-06666-7

**Published:** 2025-06-23

**Authors:** Shaimaa Ibrahim Gafar, Hany Hamza, Ihab Abdelaziz, Rania G. Estawro, Shaymaa Hassan Salah

**Affiliations:** 1 Memorial lnstitute of Ophthalmology, Cairo, Egypt; 2 Ophthalmology department, Faculty of Medicine, Cairo University, Cairo, Egypt; 3 Watany Eye Hospital, Cairo, Egypt

**Keywords:** MacTel-2, OCT-A in Mac Tel-2, Neovessels in MacTel-2, Retino-retinal anastomoses, Medical research, Macular degeneration

## Abstract

Little documented studies regarding analysis of macular telangiectasia type 2 in Egyptian patients. This prospective study included 24 eyes with MacTel-2, and 24 control eyes. Spectral-domain optical coherence tomography Angiography (OCT-A) was performed. Staging of eyes with MacTel-2, quantification of Foveal and parafoveal vascular density and full retinal thickness were performed. In MacTel-2 eyes, the superficial capillary vessel density was significantly decreased in the temporal, superior and inferior para-foveal area (p-value = 0.004, < 0.001 and 0.002 respectively). Only temporal parafoveal deep vessel density showed significant decrease in the MacTel-2 group as compared with the normal control group (49.6% versus 54.01%, p value = 0.010). There was a statistically significant decrease in the foveal and all the para-foveal thickness as compared with normal control group (*P* < 0.001). One-fourth of cases were classified as stage 4 MacTel-2. The patterns of neovessels were sea-fan, tangled or dead tree network, equally distributed among the six affected eyes. A significant reduction in both foveal density and visual acuity was documented with disease progression. This study emphasizes the role of OCT-A in diagnosis, staging and identification of neovascular pattern of MacTel-2. Furethermore, this study is considered one of the few Egyptian studies in staging the disease providing correlation to visual acuity, macular thickness and vessel density affection.

## Introduction

Macular telangiectasia type 2 (MacTel-2) is a bilateral asymmetrical macular disease caused by defects in neuroprotective function of Muller cells leading to abnormal ectatic vessels initially in deep capillary plexus^1^. Gass and Oyakawa in 1982 introduced the old terminology based on clinical data: idiopathic juxta foveolar retinal telangiectasia^2^. The staging of the disease was updated by Gass and Blodi in 1993 based on abnormalities in macular vessels into clinical five stages^3^. In 2006, based on clinical, Fluorescein angiography (FA) and Optical coherence tomography (OCT), Yannuzzi described newer stages of macular telangiectasia: non proliferative and proliferative perifoveal telangiectasia which is now known as idiopathic macular telangiectasia type 2 (MacTel-2)^4^. Clinical findings include blunting of foveal reflex, retinal graying, ectatic retinal capillaries, superficial retinal crystalline deposits, right angled venules, always start at the temporal juxta foveal area of the macula^5^. Advanced stages show retinal pigment plaques and subretinal neovascular membranes^6^. The role of FA is to detect the leaking telangiectatic vessels in the initial stages of the disease and the leaking choroidal neovessels in the late stages^3,4^. The use of OCT has deepened our understanding of MacTel-2 by quantitatively analyzing the structural changes in different disease stages. Outer retinal thinning with ellipsoid zone interruption were considered early OCT features followed by intraretinal cavitations and internal limiting membrane drape^7^.

The introduction of optical coherence tomography angiography (OCT-A), as one of the vascular imaging tools, has allowed us visualization of both superficial and deep capillary plexuses. This has been considered a huge leap in staging and monitoring MacTel-2. In the initial stages of MacTel-2, capillary dilatations (telangiectatic vessels) occur at the level of both deep and superficial capillary plexuses at temporal juxta foveal area. Chen et al. who first noted the characteristic outer retinal abnormal vascular invasion along the course of the disease, ending by subretinal neovascular membranes and scar formation in advanced stages. Chen et al. pave the way to new MacTel-2 OCTA grading system^8^. In 2016, Toto et al., re-classified the MacTel-2 using OCT-A into four simpler grades^9^. Some studies referred to the appearance of a new complex capillary network invading the outer nuclear layer, being avascular in normal eyes, and is called intraretinal abnormal vessels that appear clearly in outer retinal slab by OCT-A^10–12^. These vessels usually invade the choroid underneath ending up with the proliferative stage of MacTel-2 which is characterized by choroidal neovascular membrane (CNVM). OCT-A was successively used to point at the presence of retino-choroidal anastomoses preceding the CNVM as suggested by some publications^12–14^. Saojie et al. showed anti-vascular endothelial growth factor (Anti-VEGF) can improve visual outcome in non-proliferative MacTel-2 even with presence of abnormal outer retinal neovessels^15^.

The purpose of this research is to document the vascular and structural changes at 3 × 3 mm foveal area using OCT-A in MacTel-2 Egyptian patients. Moreover, to distinguish the neovascular patterns from ectatic vessels in the outer retinal slabs, especially in late stages of MacTel-2. Correlation of macular vessel density, macular thickness and visual acuity with disease progression was also documented in this study.

## Methods

This study is a prospective case-control analytical study. The study followed the ethical standards set out in the Declaration of Helsinki, and written informed consent was taken from all participants (patients and control) with Institutional Review Board/Ethics Committee approval (MD-221–2019). We examined twenty-four eyes of fifteen patients with MacTel-2 and twenty-four eyes of thirteen age-matched healthy controls from outpatient eye clinic in Kasr AlAiny Hospital, Cairo University from December 2019 to December 2021. Three medical institutions in Egypt participated in the clinical recruitments, however, the final assessments were done in Kasr Al Ainy Ophthalmology department, as the main study center. Eyes with MacTel-2 included in this study were selected according to guidelines by Gass and Blodi: Clinical loss of retinal transparency, presence of retinal crystalline deposits, right-angled retinal venules, pigment clumps and/or subretinal neovessels^3^. This was done by two senior Retina consultants (H.H and S.H.S). If the two consultants didn’t agree on diagnosis, the case was excluded. Any eye with macular affection due to systemic or local disease, media opacity, previous trauma and/or ocular surgery was also excluded from study.

All eyes (patients and participants) underwent ophthalmic examination in the form of visual acuity (VA) using decimal then converted to logarithmic for statistical purposes. Anterior segment examination using slit-lamp, intraocular pressure measurement using Topcon (CT-80) non-contact air-puff and posterior segment examination through fully dilated pupil using slit-lamp biomicroscopy and 90 D Volk lens. MacTel-2 eyes then underwent color photography to document clinical diagnosis using multicolor photos (SPECTRALIS^®^, Heidelberg Engineering GmbH, Germany, version:6.3.4).

### Optical coherence tomography angiography

All patients and controls underwent macular 3 × 3 mm OCT and OCT-A using a commercial spectral domain OCT system (Avanti RTVue-XR 100, Optovue Inc, Fremont, CA, Egypt) within one week of clinical diagnosis. Pupils were properly dilated with 1% tropicamide before image capture. Macular 3 × 3-mm scans including en face and structural OCTA images centered on the fovea were obtained with a split-spectrum amplitude-decorrelation angiography algorithm (AngioVue software, version 2017.1.0.151; Optovue, Inc). This machine can obtain volumetric macular scans at 70 000 scans/second speed. Both the en face and the B-scan data were only saved and analyzed after projection artifact removal and when the signal strength is better than 7/10. The software automatically segmented the full-thickness retinal scans into four slabs: the inner and middle slabs containing the superficial and deep inner retinal vascular plexuses respectively, outer retina and choriocapillaris slabs that should be avascular, were useful in identifying outer and sub-retinal neo-vascular proliferations respectively. We used custom 20-microns slab to move forward and backward to detect origin and extent of neo-capillary and any corresponding blood flow in B-scan. Vessel density (%) was automatically measured in 1-mm (foveal), 3-mm (parafoveal) circles of Early Treatment Diabetic Retinopathy Study (ETDRS) grid. FAZ; foveal avascular zone was also automatically identified and all FAZ metrics were calculated (area in mm^2^, perimeter in mm, AI; Acircularity index ratio between the measured perimeter and the perimeter of the same size circular area and FD; Foveal density within 300 μm width ring surrounding the FAZ, presented in percentage. Two experienced Retina and imaging consultant and specialist (R.E and S.I.G) evaluated the structural and MacTel-2 staging subjectively. Retinal layer thickness was evaluated on the same 3 × 3-mm OCTA acquisitions as those used for the vessel analysis. The OCT software automatically segments retinal layers and provides retinal thickness values from the ILM to the Bruch membrane. Retinal thickness is measured within foveal area and in different quadrants (temporal, superior, nasal, and inferior) of the para foveal area.

### Statistical analysis

Data was coded and entered using the statistical package for the Social Sciences (SPSS) version 26 (IBM Corp., Armonk, NY, USA). Data was summarized using mean and standard deviation for quantitative variables and frequencies (number of cases) and relative frequencies (percentages) for categorical variables. Comparisons between groups were done using unpaired t test (Chan, 2003a). For comparing categorical data, Chi square (χ^2^) test was performed. Exact test was used instead when the expected frequency is less than 5 (Chan, 2003b). Correlations between quantitative variables were done using Spearman correlation coefficient (Chan, 2003c). P-values less than 0.05 were considered statistically significant. Interobserver agreement between the two ophthalmologists for staging the MacTel-2 group was calculated using Cohen’s kappa (κ) test.

Sample size calculation used: Assuming α = 0.05 (two-tailed), Power (1-β) = 0.85, a total sample size of 48 patients, that will be allocated equally into two groups (24 per group), is required to detect an effect size (d) of 0.9 in the change between the two study groups with a power of 86.25%. Estimation of sample size was performed for t tests of means of two independent groups using computer program G * Power 3.1.9 (Franz Faul, Universität Kiel, Germany).

## Results

Our study included 48 eyes of Egyptian patients divided into two groups, the first group 24 eyes (10 right, 14 left) in patients with MacTel-2 and the second group 24 eyes (13 right, 11 left) of normal age matched control group. MacTel-2 group included 15 patients, eight were males (53.3%) and seven were females (46.7%) without statistically significant difference with the control group (p-value = 0.102). The mean age in MacTel-2 group was 62.4 years ± 1.9, the mean age in the normal group was 56.15 years ± 2.21 showing no statistically significant difference between the two groups (p-value = 0.17). The mean LogMar visual acuity in MacTel-2 group was 0.45+/−0.12 (Snellen Equivalent = 6/18) while the mean was 0.10+/−0.13 in normal group.

**Fig. 1:**
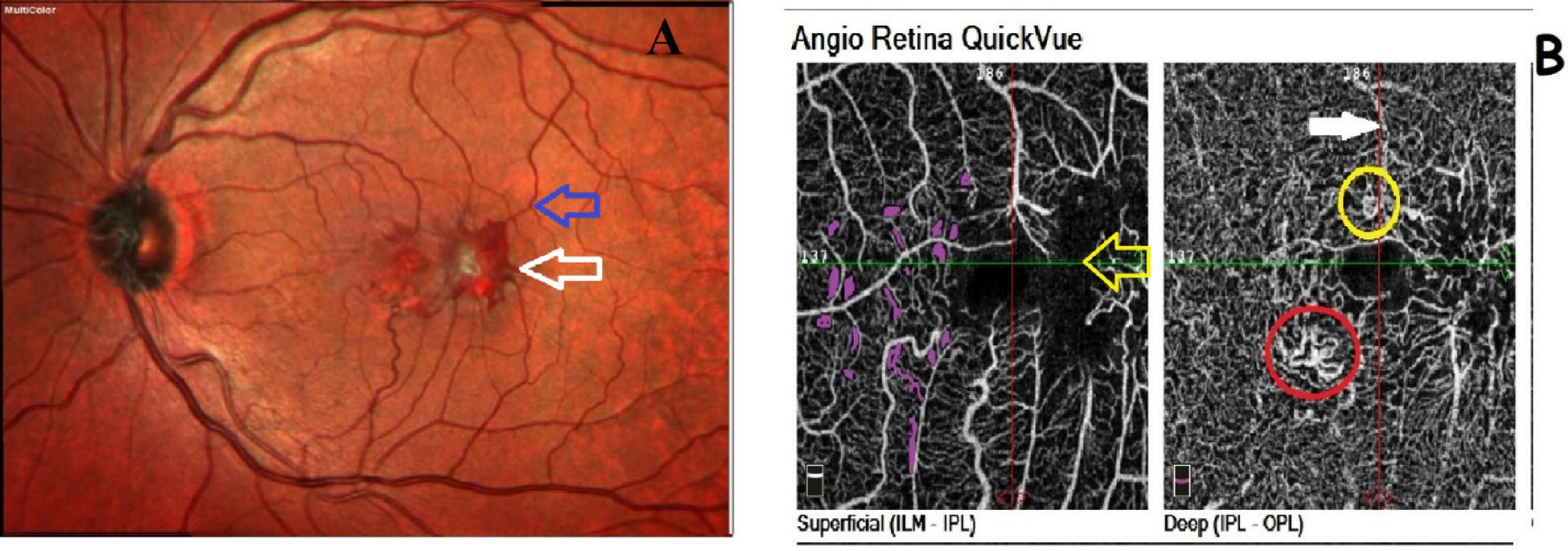
Multicolor fundus photography and Optical Coherence Tomography Angiography in the left eye of a MacTel-2 patient. (**A**) Blue empty arrow shows RAV that appeared as dilated vessel that suddenly dips into superior fovea without narrowing in caliber. White empty arrow shows an intraretinal hemorrhage. (**B**) Superficial capillary plexus SCP (left), Deep capillary plexus DCP (right) in left eye with MacTel-2 showing: Major vascular projection artifact in DCP (white filled arrow), minor vascular changes as nodular anastomosis (yellow circle), and dilatation with looping (red circle). All minute vascular changes are prominent mainly in the DCP. SCP showed mainly the major RAV and intervascular rarefactions (purple filling). Masking effect of intraretinal hemorrhage causing signal void area (yellow arrow).

In MacTel-2 eyes, 3 × 3 mm OCT-A scans showed common morphological features: Ectatic and dilated capillaries mainly in DCP, inter-vascular rarefaction mainly in SCP and DCP that should not be confused with masking artifacts of intraretinal cysts, pigmentations or hemorrhages. Right-Angled Vessels (RAVs): dilation and apparent discontinuation of blunted vessel caused by a vertical deviation from superficial to deeper retinal layers, (Fig. 1).

**Fig. 2:**
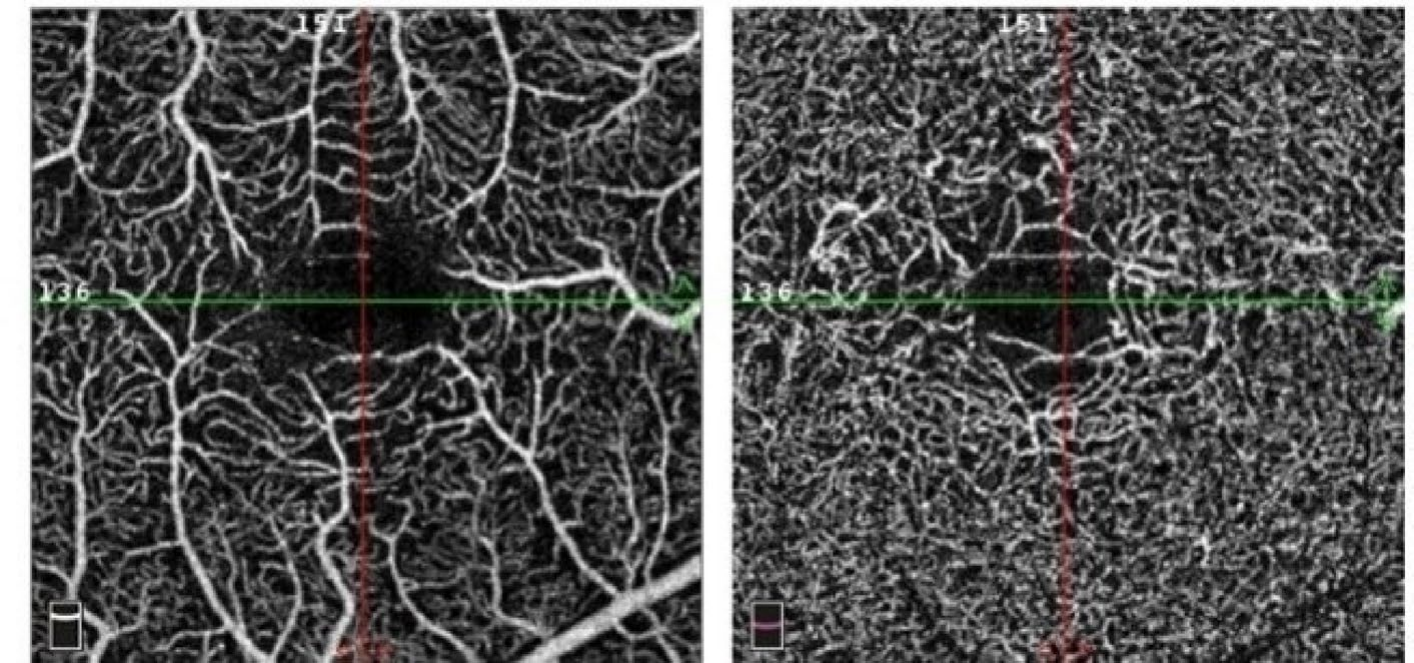
Optical Coherence Tomography Angiography of both superficial capillary plexus SCP (left) and deep capillary plexus DCP (right) in MacTel-2 Stage 3; Rarified and telangiectatic vessels circumferentially surrounding the fovea of right eye.

MacTel-2 eyes were classified according to Toto et al. classification^9^ depending on topographic localization of the telangiectatic vessels, Figure 2. Of the 24 eyes of the MacTel-2 group **8** eyes were in stage one (33.3%), **5** eyes in stage two (20.8%) **5** eyes in stage three (20.8%), **6** eyes in stage four (25%) with outer retinal and/or choroidal new vessels, (Table 1). The strength of interobserver agreement (between ophthalmologist 1 and 2) was excellent for stage 4 (κ = 0.83), and lower but still acceptable for early stages (1 to 3; κ = 0.72).

**Fig. 3:**
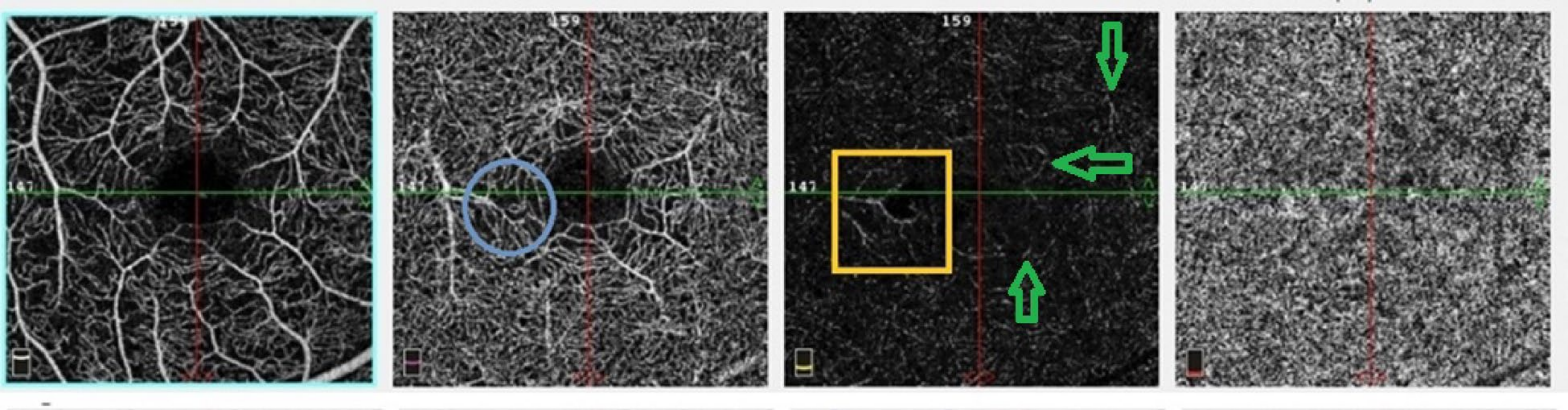
The right eyes of Macular Telangiectasia type 2 (MacTel-2) (from left to right): Superficial capillary plexus, deep capillary plexus, outer retinal slab, and choriocapillaris slab (both normally avascular). The yellow box in outer retinal slab showed: Dendritic capillaries at temporal fovea that are continuous in pattern and similar in distribution as those at same place in DCP (blue circle). Green arrows in outer retinal slab show projection artifacts of deep retinal vessels.

**Fig. 4:**
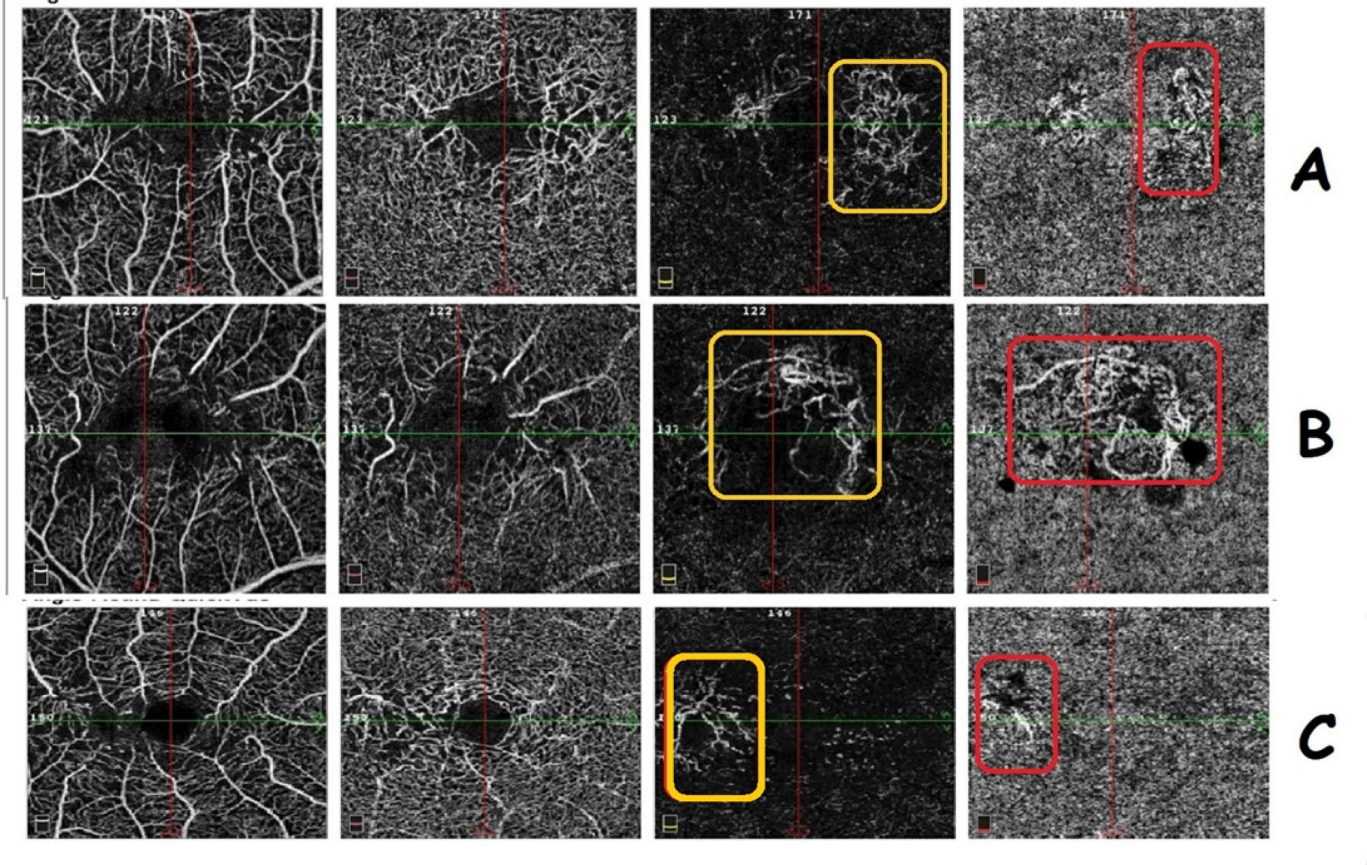
Macular Telangiectasia type 2 (MacTel-2) (from left to right): Superficial capillary plexus, deep capillary plexus, outer retinal slab, and choriocapillaris slab (last two slabs are normally avascular). Evidence of neovessels in outer retinal slab (yellow rectangle) and in choriocapillaris (red rectangle), with completely different pattern compared to deep retinal slab. Patterns of neovessels appear as temporal sea-fan in a left eye (**A**), tangled in a left eye (**B**), and dead tree in a right eye (**C**).

In MacTel-2 group, not all dilated ectatic capillaries in outer retinal slab are neovessels. By establishing the custom en-face slab, analysis of the connectivity of those capillaries with DCP and their pattern was accessible. Hence, some capillaries at that level were just dilated, branching ectasia of DCP as a common finding in non-proliferative MacTel-2 (stages 1,2,3) others have different pattern and distribution making them more prone to being retino-retinal or retino-choroidal anastomoses (stage 4), (Fig. 3). All the 6 eyes in stage 4 (100%) showed outer retinal neovessels while 4 eyes (4/6, 66.6%) showed both outer retinal and choroidal neovessels. The patterns of neovessels were sea-fan, tangled or dead tree network, equally distributed among the six eyes of stage 4, (Fig. 4). Two eyes (33.3%) in stage 4 had history of intravitreal injections, those 2 eyes had the dead tree vascular network.

**Table 1:** OCT-A classification in MacTel-2 group (24 eyes) using Toto et al. grading system.

Stage	Finding by OCT-A	Number of eyes (%)	Average BCVA by Log Mar (min-max)
One	Vascular anomalies in the deep and/or superficial plexus **temporal** to the fovea	8 (33.3)	**0.2** (0.5 − 0.1)
Two	Vascular anomalies in the deep and/or superficial plexus **temporal and nasal** to the fovea	5 (20.8)	**0.4** (0.6 − 0.2)
Three	Markedly diffuse **circumferential** vascular anomalies in the deep and superficial plexus	5 (20.8)	**0.6** (0.8 − 0.2)
Four	**Neovascularization** in the outer retina with any OCTA signs of grade 1 to 3	6 (25)	**0.8** (1.0-0.7)

FAZ measurements regarding area, perimeter, acircularity index and density showed no statistical difference between normal and MacTel-2 group. In MacTel-2 group, the vessel density in the SCP was significantly decreased in the temporal, superior and inferior para-foveal area (p-value = 0.004, < 0.001 and 0.002 respectively). However, only the temporal parafoveal vessel density in DCP showed significant decrease in the MacTel-2 group as compared with the normal control group (49.6% versus 54.01%, p-value = 0.010). In MacTel-2 group, comparison between the vessel densities in both SCP and DCP shows more significant decrease in the superficial vessel density in the foveal area and all para-foveal areas (p-value < 0.001).

Regarding retinal full thickness at the 3 × 3 mm scan, there was statistically significant decrease in the foveal and all the para-foveal thickness as compared with normal control group (Table 2).

**Table 2:** Comparison between FAZ measurements, foveal and Para foveal vessel density and macular thickness in normal and Mac Tel 2 groups.

FAZ measurements	Normal		MacTel-2		*P*-value
	Mean	SD	Mean	SD	
FAZ area in mm^2^	0.34	0.1	0.36	0.2	0.6
Perimeter in mm	2.31	0.39	2.45	0.78	0.47
AI	1.13	0.04	1.19	0.17	0.08
FD %	51.8	2.55	49.56	5.6	0.08
Superficial Vessel Density					
Foveal	15.37	7.30	16.85	8.00	0.507
Temporal parafoveal	47.98	2.78	42.97	7.46	0.004*
Superior parafoveal	50.98	3.28	46.43	4.85	< 0.001*
Nasal parafoveal	48.08	3.75	45.33	5.74	0.056
Inferior parafoveal	50.89	3.88	46.56	5.29	0.002*
Deep Vessel Density					
Foveal	30.45	6.92	29.05	10.39	0.587
Temporal parafoveal	54.01	3.47	49.60	7.10	0.010*
Superior parafoveal	52.78	4.01	52.99	4.85	0.891
Nasal parafoveal	53.65	3.16	52.48	5.74	0.419
Inferior parafoveal	53.41	3.42	54.14	5.46	0.581
Macular thickness					
Fovea	243.6	21.24	220.06	37.3	0.01*
Temporal parafoveal	308.04	14.09	272.75	38.02	< 0.001*
Superior parafoveal	322.79	18.56	290.04	33.16	< 0.001*
Nasal parafoveal	320.63	15.25	293.79	36.39	0.002*
Inferior parafoveal	318.63	13.38	282.87	21.18	< 0.001*

FAZ: Foveal Avascular Zone, AI: acircularity index, FD: Foveal Density, SD: Standard deviation, *P < 0.05 is considered statistically significant by Mann Whitney test.

Our study showed reduced foveal and para foveal vessel density in more advanced MacTel-2 stages. However, this relation was statistically insignificant in either superficial or deep plexuses. The foveal vessel density was the only significantly reduced parameter in disease progression (p-value = 0.009). Also, positive non-significant correlation was found between macular thickness and disease staging.

Visual acuity is reduced with more reduction in vessels density and with increase in macular thickness but again these correlations were non-significant. Visual acuity was significantly reduced with disease progression (p-value = 0.001).

## Discussion

Optical Coherence Tomography angiography has been the standard diagnostic non-invasive imaging tool in many retinal vascular diseases for a decade. It has a better ability to visualize the DCP that is poorly seen by conventional Fluorescein Angiography (FA)^9^. The pathophisiology of MacTel-2 is still controversial, however some researches proposed theories as neurodegeneration of Muller cells, followed by photoreceptor apoptosis and capillary dilatation ending with new capillary proliferations^13,16,17^.

In the current study the high-resolution 3 × 3 mm scans of OCT-A of 24 eyes in patients with Mac Tel-2 have been studied. We found common vascular morphological features in the eyes of the diseased group as dilated capillaries in DCP (telangiectasia), vascular rarefaction in SCP and RAVs. These features were characteristically found in all eyes with MacTel-2 regardless the stage. OCT-A has a major role in early detection of pathognomonic RAVs other than fundoscopy and FA in eyes with early stages of MacTel-2^18^.

Severe density reduction was documented in SCP in temporal, superior and inferior parafoveal areas in the MacTel-2 group (p-value = 0.004, < 0.001 and 0.002 respectively) when compared with normal control. This goes along with previous similar studies^9,19–22^. This can be attributed to the vascular rarefaction that was more evident in all eyes (100%) predominantly in the SCP rather than the deep one. In DCP, there was only significant decrease in the temporal para-foveal area in the Mac Tel group (p-value = 0.010). This however goes along with Runkle et al.^17^ that also proved affection of temporal parafoveal density in the DCP. This vascular layer was debatable in studies as Toto et al.^9^ and Ersoz et al.^21^ that showed no significant decrease in deep vascular density. On the other hand, other studies showed significant decrease in parafoveal density in DCP^23,24^. Algorithmic contradictions or differences in severeity of included eyes may play role in this debait. The loss of vascular density in SCP reflects the loss of vascular network but nearly preserved density in DCP reflects more telangiectatic vessels at that level. Our study revealed that the FAZ area, perimeter, AI and vessel density were nearly similar between eyes with MacTel 2 and healthy eyes.

We classified Mac Tel-2 into four simple stages guided by Toto et al. study^9^. This classification considered any neovessels at outer retinal slab in OCT-A as stage 4 MacTel-2. We ensured in this work the importance of custom slab movement to differentiate ectatic dilated telangiectasia from neovessels. All the 6 eyes in stage 4 (100%) showed outer retinal neovessels while 4 eyes (66.6%) showed both outer retinal and choroidal neovessels. We have studied the pattern of those neovessels and were in the form of tangled, seafan and dead tree. This was studied before in 2015 by Gaudric et al. but the study evaluated only the retino-retinal and retino-choroidal anastomoses and excluded the actual active neovascular membranes. The development of outer retinal neovessels (in outer nuclear layer) has been proved in many previous researche to be newly formed layer with different vascular pattern to the DCP (outer plexiform layer)^9,11–13^. Retinal subsidence, as described by Spaide et al. in 2018, involved the descent of deep dilated capillaries into RPE allowing more proliferation potentiality^25–27^. Those capillaries invading the outer retinal layers were either retino-retinal, retino-choroidal anastomoses or true forms of neovascular membranes with leaking characteristics as in age related maculopathy. In our study, non-significant reduction in density of both SCP and DCP was noticed with progression of disease, however only the foveal density was significantly reduced in stage 4.

Regarding retinal full thickness at the 3 × 3 mm scan, there was statistically significant decrease in the foveal and the para-foveal thickness in MacTel-2 group as compared with normal control group. This finding was documented in many studies^9,24^. This structural thinning is mostly observed due to Muller fibers degenerations causing outer retinal thinning. This was observed, in our study, in early stages more than late stages of the disease. As we found a non-significant increase of thickness with disease progression. Increased macular thickness in late stage (proliferative) is usually seen with outer retinal and choroidal neovessels. Studying correlation of visual acuity with vessel density and thickness in MacTel-2 group, we found a non-significant decrease in visual acuity with decreased vessel density and increased macular thickness. The visual acuity was significantly reduced with disease progression (mean value: 0.8 in stage 4 as compared to 0.2 in stage 1, Table 1). This strong correlation goes with some studies^28,29^ and was opposed by others^30^.

Limitations of this study include difficulty in image capture due to poor fixation, poor segmentation and motion artifacts that hinder recruiting more diseased eyes. Technical limitations also hinder proper acquisition of images as in MacTel-2 eyes degenerations in outer retinal layers and intraretinal pigmentations can cause a lot of projection and masking artifacts respectively.

In conclusion: OCT-A plays a fundamental role in diagnosis, staging and identification of neovascular pattern of MacTel-2. In stage 4 MacTel-2 shows reduced visual acuity, increased macular thickness and decreased foveal density.

## Data Availability

The datasets used and analysis in current study are available at corresponding author (Shaymaa Hassan Salah-Shaimaa.sayed@kasrAlainy.edu.eg) on request.
